# Supplementary material for: Bioefficacy of Nga-Mon (Perilla frutescens) Fresh and Dry Leaf: Assessment of Antioxidant, Antimutagenicity, and Anti-Inflammatory Potential
Source: Plants (Basel). 2023 Jun 3;12(11):2210. doi: 10.3390/plants12112210 (PMC10255129; doi:10.3390/plants12112210)
Supplement: Supplementary file 1 [file plants-12-02210-s001.zip › plants-2434460-supplementary.docx]

Supplementary Materials

Bioefficacy of Nga-Mon (*Perilla frutescens*) Fresh and Dry Leaf: Assessment of Antioxidant, Antimutagenicity, and Anti-inflammatory Potential

Payungsak Tantipaiboonwong^1^, Komsak Pintha^1^, Wittaya Chaiwangyen^1^, Maitree Suttajit^1^, Chakkrit Khanaree^2^, and Orawan Khantamat^3,^*

^1^ Division of Biochemistry, School of Medical Sciences, University of Phayao, Phayao 56000, Thailand

^2^ School of Traditional and Alternative Medicine, Chiang Rai Rajabhat University, Chiang Rai 57100, Thailand

^3^ Department of Biochemistry, Faculty of Medicine, Chiang Mai University, Chiang Mai 50200, Thailand

***** Correspondence: orawan.kh@cmu.ac.th

**

**

**Figure S1.** HPLC chromatographic profile of Thai perilla fresh leaf extract (PLE_f_).





**Figure S2.** HPLC chromatographic profile of Thai perilla dry leaf extract (PLE_d_).
